# The impact of exercise modalities on blood glucose, blood pressure and body composition in patients with type 2 diabetes mellitus

**DOI:** 10.1186/s13102-023-00762-9

**Published:** 2023-11-14

**Authors:** Tensay Ambelu, Getu Teferi

**Affiliations:** Department of Sport Science, Debre Markos University, Debremarkos, Ethiopia

**Keywords:** Type 2 Diabetes Mellitus, Body composition, Fastingg blood glucose, Blood pressure, Resistance, And aerobic training

## Abstract

**Background:**

Physical activity has been recommended as an important non-pharmacological therapeutic strategy for the management of type 2 diabetes mellitus (T2DM). The aim of this study was to investigate the effects of 12 weeks of strength, aerobic, and a combination of aerobic and resistance training on blood glucose level, blood pressure, and body composition in patients with T2DM.

**Methods:**

From Debremarkos referral hospital, 40 subjects with T2DM (mean age 42.45 years, 29 men, 11 women) were randomly assigned to one of three intervention groups or the control group. The following variables were measured: body mass index (BMI), fasting blood glucose (FBG), systolic blood pressure (SBP), diastolic blood pressure (DBP), and body fat percentage (BFP). Paired sample T-test and one-way ANCOVA were applied whilst controlling for diet, gender, and age.

**Results:**

All intervention groups showed improvement in a mean difference of FBG − 13.03 (t =-5.55, df = 39, p < 0.001), SBP − 21.63 mmHg − 17.6 mmHg (t =-6.51, df = 39, p < 0.001), DBP − 11.86 mmHg (t = -5.47, df = 39, p < 0.001) and BFP − 9.14 (t = -7.49, df = 39, p < 0.001). There was a significant difference in mean BMI reduction when diet, gender, and age were controlled in a one-way ANCOVA (F (3, 33) = 11.79, p < 0.001), SBP (F (3, 33) = 13.383, p < 0.001), DBP (F (3, 33) = 7.830, p < 0.001), FBG (F (3, 33) = 6.337, p < 0.001), BFP (F (3, 33) = 24.29, p < 0.001) between the exercise intervention groups and control group. Additionally, the estimated marginal means indicate that the combined strength and aerobic exercise intervention group experienced the greatest improvements.

**Conclusion:**

Body composition, blood pressure, and fasting blood glucose were significantly lower in the combined (aerobic plus strength) treatment than in the individual treatment, indicating that the combined exercise intervention was more successful in altering these parameters.

**Supplementary Information:**

The online version contains supplementary material available at 10.1186/s13102-023-00762-9.

## Introduction

Diabetes mellitus (DM) is a group of metabolic disorder characterized by an elevated blood glucose level as a result of limitation in insulin secretion or an inability to use insulin [1]. Around 382 million adults worldwide suffer from diabetes mellitus, which is expected to reach 439 million by 2030 [2, 3] and 85–95% of diabetes cases worldwide are T2DM [3]. T2DM, with an increasing rate of prevalence in recent decades [4], has caused many health and socioeconomic problems throughout the world. Due to its various consequences and disabilities, T2DM has been known as a disabling disease, as well. Therefore, strategies for reducing the healthcare costs associated with the disease should be emphasized [5]. According to the Centers for Disease Control and Prevention, 9.3% of the U.S. population have diabetes [6]. The growing number of non-communicable diseases (NCDs) like diabetes is currently a problem in Ethiopia [7]. In Ethiopia, as per the public WHO study of 2015, the commonness of diabetes mellitus was 3.2% [7]. A systematic review on the prevalence of T2DM in Ethiopia also found that 2–6.5% [8].

One of the main risk factors for T2DM and its complications is a sedentary lifestyle [9]. As an important non-pharmacological treatment option for T2DM, physical activity has been suggested [10]. Physical activity (PA) is an important treatment for any type of diabetes and may help prevent diabetes-related health complications, insulin resistance, and T2DM [11]. Currently, many people are aware that T2DM is caused by a sedentary lifestyle [12, 13]. One of the components of wellness that is effective in treating and preventing T2DM is daily exercise or physical activity [14]. Improved blood glucose levels, increased insulin sensitivity, prevention or delay of type 2 diabetes development, and decreased glucose concentration are all benefits of T2DM patients engaging in regular exercise [15, 16].

Preventative medicine calls for lifestyle changes such as regular exercise to reduce the risk of developing these diseases [17]. Exercise can improve glycemic control and blood lipid profiles in people with and without type 2 diabetes [18, 19]. Thus, incorporating sustainable exercise modalities that improve cardio-metabolic risk factors can improve health outcomes. The impact of aerobic training (AT) on health outcomes is well researched [20]. Aerobic exercise refers to activities such as walking or jogging with continuous, repetitive movement of large muscle groups for at least 10 min at a time. Aerobic exercise may modify the insulin action of each fiber without increasing fiber size [21]. Aerobic exercise is known to manage glycemic control and cardiovascular risk factors. It has also beneficial effects for metabolic profile in patients with T2DM [22]. Aerobic (endurance) exercise increases skeletal muscle capitalization and blood flow, muscular GLUT4 levels, hexokinase, and glycogen synthase activities [23]. The American Diabetes Association (ADA) recommends at least 150 min per week of moderate-intensity aerobic physical activity or at least 90 min per week of vigorous aerobic exercise spread out over at least three days per week and no more than two days without physical activity [24]. Resistance training as been shown to induce a hypertrophic response and a muscle fiber type shift in exercising muscles, which causes an increase in whole-body glucose utilization [25]. Resistance training improves muscular strength and endurance, enhances flexibility and body composition, and decreases the risk of cardiovascular disease [26]. Possible underlying mechanisms for resistance (strength) training’s positive effects include an increase in the number of glucose transporter (GLUT) proteins, increased total muscle mass, and an increase in the number of insulin receptors in muscle cells [23]. In contrast to aerobic exercise, the ADA only began recommending resistance exercise in 2006. In the absence of contraindications, diabetic patients should be encouraged to practice resistance training three times per week, targeting all main muscle groups, progressing to three sets of eight to ten repetitions at a weight that cannot be raised more than eight to ten times. [24]. Resistance training (RT) has gained popularity for its impact on improving body composition and muscular strength and, more recently, for its role in health and disease [27]. These benefits include improved glycemic control, blood lipid profiles, and bone mineral density in healthy populations [27].

Current national and international guidelines recommend aerobic and resistance exercise training for T2DM patients [28, 29]. A combination of aerobic and resistance exercise (combined exercise) has been recommended by the European Society of Cardiology [28], American College of Sports Medicine [30], and Exercise and Sports Science Australia [31]. Research has supported the combined benefits of AT and RT to improve glycemic control and cardio-metabolic health in T2DM patients [32].

Thus, multiple exercise training modalities have been recommended by different international organizations. However, it is difficult to determine the superiority of different physical activities for T2DM [33]. Research comparing the effect of resistance and aerobic training on T2DM and its risk factors is limited and few studies have compared aerobic, resistance and a combination of this training, more research is needed to better understand the effect of different exercise modalities [34]. Therefore, the present study aimed to compare the effectiveness of different modes of exercise (aerobic, resistance training as well as combined exercise versus a control group) in the improvement of blood glucose, blood pressure, and body composition for patients with T2DM.

## Methods and materials

### Research setting and design

Participants were physically inactive, aged 18 and above years with type 2 diabetes mellitus, recruited from patients registered in the outpatient department of Debremarkos referral hospital (Debre Markos, Ethiopia). Type 2 diabetic patients (*n* = 40) were enrolled from the Diabetic Outpatient unit of Debremarkos referral hospital. Inclusion criteria were: (a) BMI > 25.0 kgm-2 (b) pre-diabetic or diabetic type 2 patients (fasting blood glucose > 126 mg/dl-1) [35] (c) volunteer to participate and (d) patients were physically inactive (not achieving 30–60 min per day or 150 min per week of moderate intensity exercise or 20–60 min per day (75 min per week) of vigorous intensity exercise [35] and cleared a medical history form (the physical activity readiness questionnaire). Exclusion criteria were: (a) any cardiovascular, respiratory, or muscle-skeletal disorders precluding physical exercise, (b) uncontrolled hyperglycemia (> 250 mg/dl) and hypertension (Resting BP > 200/115), (c) active infection (d) acute myocardial infarction, stroke, trauma, or surgery and severe liver dysfunction.

All experimental procedures, risks, and protocols were fully explained to subjects before they gave their informed consent in accordance with American College of Sports Medicine guidelines [35]. Additionally, all procedures in studies involving human participants were carried out in accordance with Debremarkos University’s department of sports science research ethics committee (approval number:SPSC03/2021).The research design of this study was reflective of a “classic design for exploring the effect of intervention training”, the pretest-posttest randomized four group parallel experimental design [36].

**Fig. 1:**
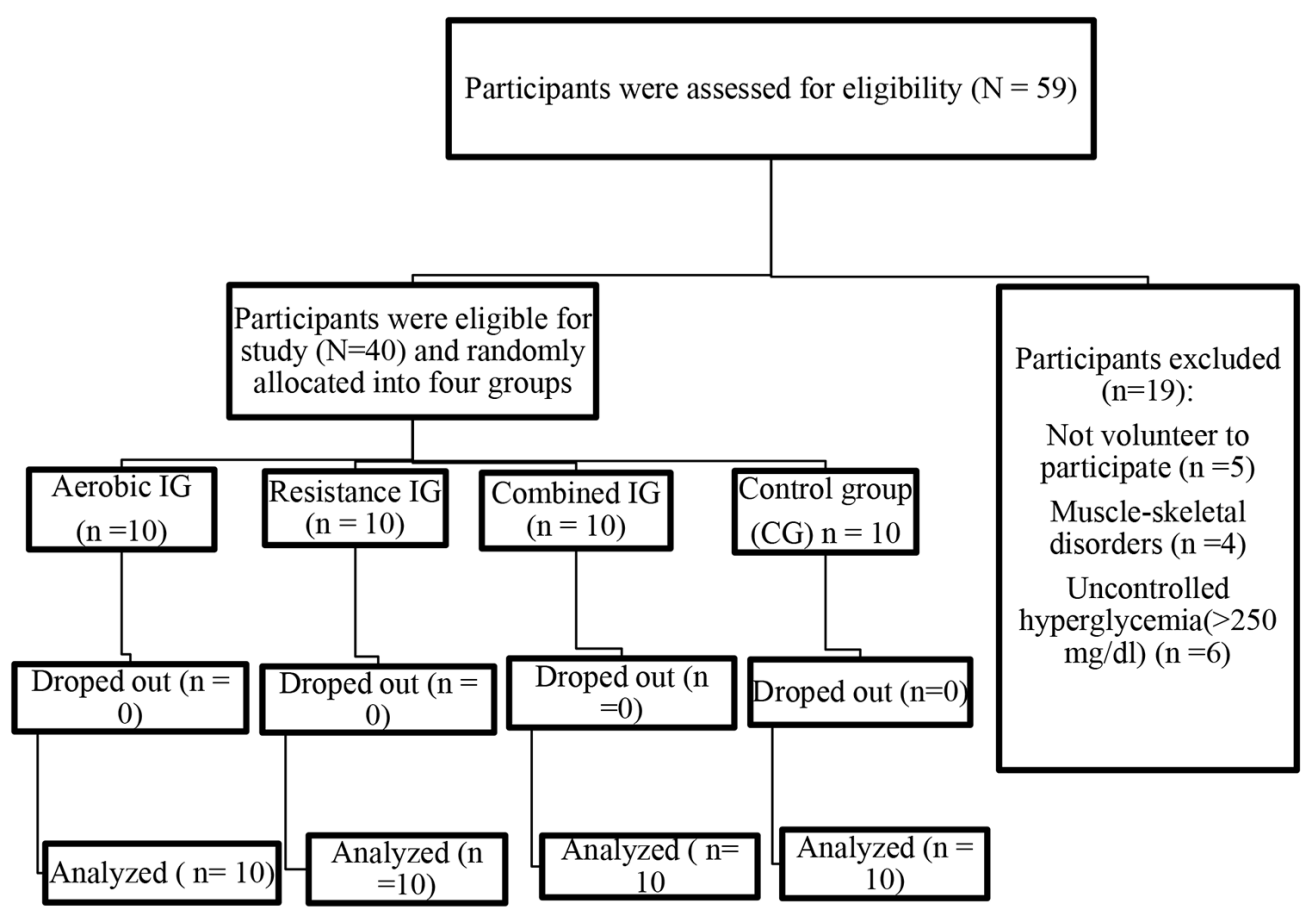
Participant flow chart

The source population of this trial pilot study was all patients with T2DM who met the inclusion criteria. Two weeks prior to the study period, patients with T2DM who visited the hospital were recruited. According to the hospital’s general outpatient medical record, six to eight patients with T2DM visited the facility each day. If we use the lower limit, which covers five days per week, this equates to approximately 30 patients per week. However, 59 patients with T2DM (totally 1 subject was less than the expected 60 subjects) were considered the target population; two weeks prior to the study period, but 19 of them were not fulfilling inclusion criteria. The remaining 40 patients with T2DM were randomly assigned to one of four groups using a random numbered table: aerobic training group (ATG: n = 10), resistance training group (RTG: n = 10), combined training group (resistance plus aerobic; CTG: n = 10) and control group (CG: n = 10), (Fig. 1). We used a simple stratified randomization method because stratification is used to ensure a good balance of participant characteristics in each group (gender). Mechanism used to implement the random allocation sequence was a random numbered table by staffs who were not assessors of the outcome. Baseline characteristics may not be well matched between study groups without stratification. Additionally, the study group to which trial participants had already been assigned was unknown. The pre-test (baseline) data were gathered prior to the intervention—also known as the baseline or pre-intervention data—while the post-test data were gathered following the 12-week intervention. The data were collected in Debremarkos referral hospital and department of sports science gymnasium. We measured blood glucose as the primary outcome and body composition and blood pressure as secondary outcomes. The periods of follow-up (intervention) was from 31 to 2021 to 31 October 2021.

### Measurement of study variables

#### Fasting blood sugar level (FBS) test

Blood glucose level at rest: The Accucheck glucometer was used to measure the subjects’ fasting blood glucose levels before and after 12 weeks of exercise training under the supervision of a medical laboratory expert. A trained medical laboratory technician took the blood sample after an overnight fast, transported it to a standard tertiary care laboratory, and then sent it to the Debremarkos referral hospital for analysis. The study’s laboratory technicians were blinded before and after the intervention.

#### Blood pressure

Blood pressure was measured with an automated Sphygmacor XCEL. The participant was seated with straight legs and a brachial pressure cuff over their left arm’s brachial artery. The device measured the brachial systolic and diastolic blood pressure three times, with a two-minute rest in between each measurement. The SphygmaCor XCEL reported the average of the last two readings instead of the initial reading.

#### Body composition

The body mass index was calculated by dividing the height in meters squared by the body weight in kilograms using a standard stadiometer (Height & Weight Scale ZT-160 – NSL). Skinfold determination of percent body fat can be quite accurate when performed by a properly trained technician with a skinfold caliper (LeTkingok: High Precision Sebum Thickness Meter Skin Fold Caliper). It is estimated that the proportion of subcutaneous to total fat varies with gender and age [37, 38]. For this study, we used the Jackson-Pollock 3-Site Skinfold formula for Body Density J-P 3-Site [39] based on the specific recommendations of [37, 38]. Thus, with sex specific body site for body fat measurement of women was three site on triceps, suprailiac and abdominal with three-site formula (triceps, suprailiac, abdominal): Body Density = 1.089733 − 0.0009245 (sum of three skinfolds) + 0.0000025 (sum of three skinfolds)^2^ − 0.0000979 (age), on the other hand for men the three- site on chest, triceps and subscapular with three-site formula (chest, triceps, subscapular): Body Density = 1.1125025 − 0.0013125 (sum of three skinfolds) + 0.0000055 (sum of three skinfolds)^2^ − 0.000244 (age). According to Siri Equation [39], the percent of body fat can be determined using Body Density (BD), as: Siri Equation % Body Fat = 495/BD – 450.

### Covariate Variable

A covariate is not part of the main research question but could influence the dependent variable and therefore needs to be controlled for. Instruments for measuring food frequency [40] were used. Three-day dietary records—2 weekdays and 1 weekend day— were taken per week in a 3-month follow-up (3 exercise groups) with control group and were analyzed using the mean of three-day dietary records through one way ANCOVA to control the influence.

### Exercise intervention protocol

During the 12-week training period, all study participants, with the exception of the non-exercise control group, exercised for 60 min, which included 10–15 min of warming up and dynamic stretching, 10–15 min of cooling down and static stretching [41], and 30–40 min main workout. All training sessions were overseen by health fitness professionals and exercise physiologists. The exercise regimens developed by the American College of Sports Medicine [42, 43] served as the foundation. The exercise interventions groups (IG) were aerobic IG, resistance IG, and combined IG were used in this study. The total time spent on each session was used to equalize the exercise protocols. The aerobic exercise-only group performed aerobic dance at an initial heart rate of 40% heart rate reserve (HRR) and gradually increased to approximately 70% HRR as the intervention progressed [35]. The heart rate monitor that was worn by the participants throughout each exercise session recorded their maximum heart rate, which could be exercised at an intensity that did not exceed 80% of their maximum heart rate. The heart rate monitor that we used was the Polar H7 heart rate monitor (Polar Electro, Kempele, Finland), which contains a single flexible plastic sensor (2.4 × 27.9 cm), worn concurrently and placed on the sternum. The Polar H7 heart rate receiver has a sample rate of 1000 Hz and has high agreement with ECG measurements during various exercise modalities [44].Calculation of the maximum heart rate was based on Gellish et al., [45]. Due to the wide age range of participants in an adult fitness program, this formula is recommended for both men and women.

The only resistance group that includes: standing plantar flexion, triceps pulley, neutral rowing, squatting, dumbbell supine, knee extension with ankle weights, dumbbell development, dumbbell curl and trunk flexion and vertical bench press [46]. The circuit type of resistance training (RT) was used with intervals of 15–20 s between exercises, with 3 sets of 10 repetitions with a rest of 1–2 min between sets. Due to the patients’ lack of physical fitness and motor coordination, the loads were determined by their perceived exertion using the scale of 6 to 20 proposed by Borg in 1982 [47]. The values used were 11 to 13, which represented a moderate effort. The load was then increased with the goal of maintaining constant value of perceived exertion. The combined group participated in 30 min of resistance training and 30 min of aerobic exercise at the same intensity, progression, and method in each session. The only difference for aerobic exercise was that it was cut down to 30 min rather than 60. With the exception of the neutral rowing, dumbbell supine, dumbbell development and standing plantar flexion, these participants performed their resistance training with the same intensity and protocol as the afore-mentioned individual groups, reducing it to six exercises instead of ten and two sets instead of three. All participants were asked not to do any moderate or vigorous physical activity outside of the intervention. During the first week of the intervention and after the 12th week, a three-day food diary was obtained.

Regarding to control methods for menstrual cycle of pre-menopausal women between testing periods, we used the calendar-based counting method combined with individualized training (for the first three days) was a control method for menstrual cycle of pre-menopausal women during the exercise intervention period. During menstrual cycle, it can be difficult to follow an exercise routine because progesterone and estrogen are at their lowest, which can result in feeling less energy and motivation. With stamina and endurance levels diminished, may not feel up to fast-paced, cardio activities or workouts that rely on lifting heavy weights [48]. However, that does not mean that exercising is not advised during this time. Consider low intensity cardio, and with light weight strength training. Even walking for their period can be beneficial. But need to exclude schedule of a harder time exercising during hot and humid weather [49, 50].

### Statistical analysis

Shapiro-Wilk test was employed to assess the normality of the distributions for all variables that were observed. The Shiapro-Wilk test is a test of normality that assesses whether a sample is likely to originate from a normal distribution [51]. Data analyses were carried out using the analyses performed using SPSS Statistics for Windows (version 24.0; SPSS Inc., Chicago, IL, USA). Descriptive statistics, such as mean and standard deviations and inferential statistics: the Paired sample T-test was used to describe the pre and post-tests of study variables and one way, ANCOVA, was employed to identify the potential intervention exercise group by comparing means between groups and to control covariate variables of diet, gender and age. Subsequently, when the ANOVA results were significantly different, we used Bonferroni’s correction as a multiple comparison test. All statistical levels of significance were to be set at the “p” value ≤ 0.05.

## Results

### Characteristics of subjects

This study included forty participants (29 male, 11 women), all of whom completed the exercise program. The gender distribution across four groups was almost equal; male 70% and women 30% in Aerobic IG, Resistance IG and Combined IG but 80% men and 20% women for control group. It was observed that the participants were challenged to finish a workout session without resting for a few minutes at the beginning of the intervention. However, most participants were able to finish the exercise intervention after the sixth week. The study variables did not differ significantly prior to the intervention, as shown in Table 1. Additionally, normality test of data (Shapiro-Wilk Test) across four groups revealed that the data was normally distributed (Table 1). Mean of age in strength IG (42.30), aerobic IG (41.10), combined IG (42.6) and control group (43.80). Mean of fasting blood glucose level in strength IG (154.6), aerobic IG (151.3), combined IG (154.4) and control group (150.1). Mean of body fat percentage in strength IG (39.22), aerobic IG (39.8), combined IG (39) and control group (39), all had the same mean percentage of body fat. In this instance, it appears that the randomization of the samples into four groups has no significant effect on the intervention and the data was normally distributed.

**Table 1:** Baseline data and normality test

Variables	Type of groups	Mean (SD)	Normality Test (Shapiro-Wilk Test)	
			Statistics	P-value
Age, Years	Aerobic IG	41.10 (7.218)	0.964	0.829
	Resistance IG	42.30 (4.5)	0.948	0.647
	Combined IG	42.60 (7.199)	0.837	0.041
	Control Group	43.80 (5.86)	0.853	0.063
BMI, kg/m2	Aerobic IG	28.45 (3.67)	0.908	0.267
	Resistance IG	30.01 (3.3)	0.901	0.225
	Combined IG	29.26 (2.94)	0.932	0.469
	Control Group	27.32 (3.44)	0.933	0.470
FBG	Aerobic IG	151.3 (16.1)	0.830	0.034
	Resistance IG	154.6 (11.33)	0.907	0.264
	Combined IG	154.4 (15.33)	0.886	0.152
	Control Group	150.1 (9.52)	0.887	0.153
SBP, mmHg	Aerobic IG	153.7 (19.14)	0.822	0.027
	Resistance IG	151.9 (7.65)	0.971	0.898
	Combined IG	154.1(16.45)	0.883	0.143
	Control Group	150.3 (12.27)	0.883	0.143
DBP,mmHg	Aerobic IG	99.1 (8.478)	0.843	0.049
	Resistance IG	100.6 (14.58)	0.932	0.465
	Combined IG	103.2 (12.78)	0.980	0.966
	Control Group	103.2 (12.78)	0.980	0.966
BFP, %	Aerobic IG	39.8 (4.54)	0.890	0.171
	Resistance IG	39.22 (5.58)	0.888	0.161
	Combined IG	39 (3.09)	0.908	0.267
	Control Group	39 (3.09)	0.908	0.267
Diet, calorie	Aerobic IG	2643.4 (102.15)	0.877	0.121
	Resistance IG	2655.8 (97.4)	0.827	0.031
	Combined IG	2636.2 (93.06)	0.912	0.298
	Control Group	2666.5 (144.612)	0.912	0.298

Data presented as mean (SD) for continuous variables and number and percentage for gender

Note: IGs = Intervention groups, BMI = Body mass index, FBS, Fasting blood glucose, SBP = Systolic blood pressure, DBP = Diastolic blood pressure, BFP = Body fat percentage

**Fig. 2:**
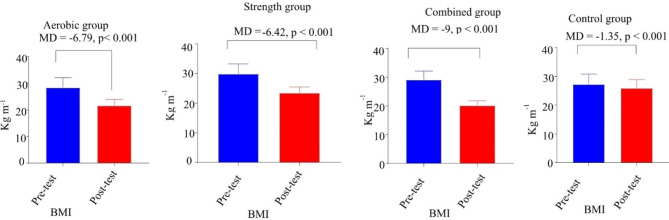
paired sample t-test of body mass index in aerobic, strength, combined and control group

**Fig. 3:**
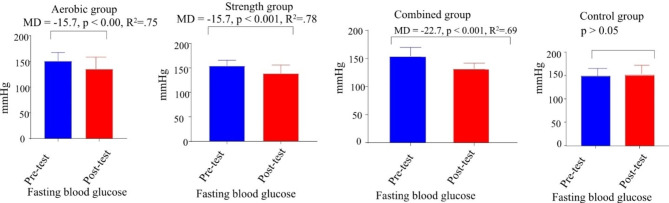
paired sample t-test of fasting blood glucose in aerobic, strength, combined and control group

**Fig. 4:**
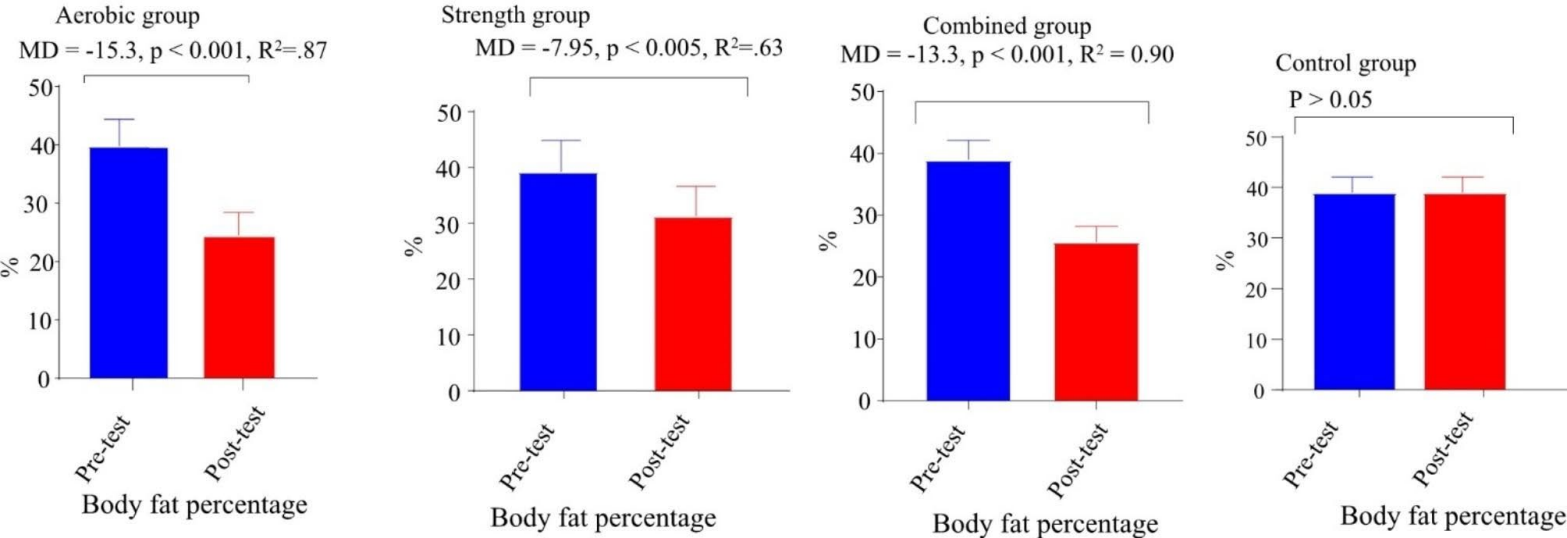
Paired sample t-test of percent body fat in aerobic, strength, combined and control group. **Key**: Figures 2, 3 and 4 have presented changes of BMI, fasting blood glucose and BF before and after 12 week interventions through paired sample t-test in aerobic, strength, combined and control groups; MD: mean difference, R^2^: effect size (eta squared)

Paired t-test was used to compare intragroup changes in BMI, FBG, and %BF (Figs. 2, 3 and 4). The results of paired t-test showed that there was a statically significant (p < 0.005) difference between the pre-test and posttest of study parameters in all intervention groups but there is no significance difference in control group (Figs. 2, 3 and 4). Additionally, the paired t-test showed significance differences between pre-test and posttest in SBP t (9) = -3.659, MD = -16.1mmHg, p < 0.005 and DBP t (9) = -10.06, MD = -11.9 mmHg, p < 0.005 for strength group; SBP t (9) = -6.478, MD = -33.3mmHg, p < 0.001 and DBP t (9) = -4.945, MD = -22.73, p < 0.005 for combined group and SBP t (9) = -5.01, MD = -20.5, p < 0.005 and DBP t (9) = -2.99, MD = -12.8mmHg, p < 0.05 for aerobic group. Generally, there was a statistically significant difference between the pretest and post-test of all measured variables in all three distinct intervention training protocols at (p < 0.005).

The primary objective of this article is to determine the most effective intervention modalities to enhance the study variables. An ANCOVA with Post hoc test was used to determine which training intervention had the greatest impact. There were significant differences of three types of intervention training comparing with control group for FBG (F = 8.08, df = 3, p < 0.001), SBP ( F = 15.24, df = 3, p < 0.001), DBP (F = 10.68, df = 3, p < 0.001), BMI ( F = 12.32, df = 3 and p < 0.001), and BFP (F = 29.11, df = 3, p < 0.001).

In addition, multiple group pairwise comparisons of the post hoc test of FBG, SBP, DBP, BMI, and BFP mean differences between the three interventions and a control groups are displayed in Table 2. All three intervention training groups have shown a reduction in blood pressure, blood glucose and body composition, however, the most effective intervention training was combined resistance plus aerobic training. The combined IG vs. control group showed significant differences (p < 0.05) with reductions in BMI (-4.32), FBG (-16.5mmHg), SBP (-24.9 mmHg), DBP (-22.73mmHg), BFP (-13.3%). Generally, combined training more significantly reduced BMI, FBG, SBP, DBP and BFP compared to the control group. However, diet has not significant difference across intervention groups.

A one-way ANCOVA was used to control for diet, gender, and age to compare the effects of three exercise interventions on BMI, FBG, SBP, DBP and BFP. The assumptions were met, and Levene’s test and normality checks were carried out. The mean reduction in BMI was significantly different [F (3, 33) = 11.79, p < 0.001], SBP [F (3, 33) = 13.383, p < 0.001], DBP [F (3, 33) = 7.830, p < 0.001], FBG [F (3, 33) = 6.337, p < 0.001], BFP [F (3, 33) = 24.29, p < 0.001] between the exercise intervention groups.

**Table 2:** One way ANOVA multiple group comparison of FBG, BFP, SBP, DBP and BMI among type 2 patients

Variables	Intervention Groups		MD	SE	P-value	95 % Confidence Interval of the Difference	
	(I) Type of groups	(J) Type of groups	(I-J)			Lower	Upper
BMI	Aerobic IG	Control Group	-4.32*	1.00	.002	-7.25	-1.39
	Resistance IG		-2.38	1.00	.149	-5.31	.55
	Combined IG		-5.72**	1.00	.000	-8.65	-2.79
	Combined IG	Resistance IG	-3.34*	1.00	.020	-6.27	− .41
FBG	Aerobic IG	Control Group	-16.5*	4.41	.007	-29.42	-3.58
	Resistance IG		-13.20*	4.41	.044	-26.12	− .28
	Combined IG		-20.4*	4.41	.001	-33.32	-7.48
SBP	Aerobic IG	Control Group	-16.6*	4.31	.006	-29.24	-3.96
	Resistance IG		-14.00*	4.31	.025	-26.64	-1.36
	Combined IG		-24.9**	4.31	.000	-41.64	-16.36
	CTG	RTG	-15.00*	4.31	.014	-27.64	-2.36
DBP	Aerobic IG	Control Group	-16.9*	4.18	.003	-29.15	-4.64
	Resistance IG		-14.5*	4.18	.015	-26.76	-2.24
	Combined IG		-22.73*	4.18	.000	-34.99	-10.47
BFP	Aerobic IG	Control Group	-14.5*	1.73	.000	-19.58	-9.42
	Resistance IG		-7.74*	1.73	.001	-12.82	-2.65
	Combined IG		-13.3*	1.73	.000	-18.38	-8.22
	Aerobic IG	RTG	-6.77*	1.73	.005	-11.84	-1.68
	Combined IG		-5.57*	1.73	.027	-10.65	− .48
Diet	Aerobic IG	Control Group	-71.5	51.7	.596	-223.16	80.17
	Resistance IG		-47.6	51.7	.838	-199.26	104.07
	Combined IG		-67.00	51.7	.645	-218.67	84.67

Key: *: significant at p < 0.05, **: significant at p < 0.01

Furthermore, examination of the estimated marginal means reveals that, in comparison to the control group, the combined strength and aerobic exercise intervention group has shown more improvements (Table 3)

**Table 3:** Estimated marginal means by controlling diet, gender and age

Dependent Variables	Type of IG	Estimates		95% Confidence Interval	
		Mean	SE	Lower Bound	Upper Bound
BMI	Strength IG	23.56	0.71	22.13	24.99
	aerobic IG	21.53	0.72	20.08	22.99
	Combined IG	20.26	0.71	18.82	21.7
	CG	26.11	0.74	24.61	27.61
FBG	Strength IG	138.9	3.25	132.29	145.50
	aerobic IG	135.75	3.3	129.03	142.46
	Combined IG	131.82	3.27	125.17	138.47
	CG	151.84	3.4	144.92	158.76
SBP	Strength IG	135.77	3.14	129.37	142.16
	aerobic IG	132.86	3.19	126.36	139.36
	Combined IG	120.83	3.16	114.39	127.27
	CG	150.14	3.29	143.44	156.84
DBP	Strength IG	88.86	2.97	82.812	94.91
	aerobic IG	87.16	3.02	81.01	93.31
	Combined IG	80.87	2.99	74.78	86.95
	CG	101.79	3.12	95.45	108.13
BFP	Strength IG	31.24	1.27	28.69	33.83
	aerobic IG	24.43	1.29	21.88	27.05
	Combined IG	25.71	1.28	23.11	28.31
	CG	39.09	1.33	36.38	41.79

According to Cohen et al., [52], for eta squared, threshold values are interpreted as small (0.01), medium (0.06), and large effects (0.14). It can be seen that for the intervention group effect size of BMI (0.52), FBG (0.38), SBP (0.55), DBP (0.42) and BFP (0.69). Those values are also used to describe how much of the variance in the dependent variable is explained by the independent variable. Ideally partial Eta Squared value of BMI, FBG, SBP, DBP, and BFP revealed that large effect (Table S1).

## Discussion

The purpose of this study was to compare the effects of a 12-week exercise program (aerobic exercise intervention group, strength exercise group, and combined aerobic and resistance exercise group on the fasting blood glucose level, body fat percentage, and blood pressure among patients with type 2 diabetes. We found that, patients with type 2 diabetes’ fasting blood glucose level, body fat percentage, and blood pressure decreased in the three intervention groups. From aerobic and strength exercise alone, the most promising exercise intervention was combined strength and aerobic exercise. According to current national and international physical activity guidelines, combined aerobic and resistance exercise training was recommended for patients with T2DM [53–56]. Our findings give support for this recommendation, as both resistance and aerobic training have a positive therapeutic effect in the treatment and control of T2DM. However, the combination of both types of training seems to have a greater impact on glycaemic control than both types of exercise alone [29, 31, 57]. Kang and Baek [58] found that the 12 weeks combined aerobic and resistance training programme reduced significantly fasting blood glucose among patients with T2DM.

Other studies that are similar to the current study found that combined training (aerobic training plus strength training) caused a decrease in fat levels around abdominal area [59], and using combined aerobic plus strength exercise was the most effective training program for fat burning [60]. Additionally, aerobic training helps to lower body fat and strength training used to increase fat-free body mass or preserved existing body mass [61]. In line with this our study also have shown that aerobic exercise intervention can more reduce body fat percentage, blood pressure and fasting blood glucose among type 2 diabetes patient than strength intervention exercise alone.

Weight problems may lead to insulin resistance, which is a risk factor to the pathophysiological mechanism of T2DM. In terms of therapy for Type 2 diabetes, it is viewed integral to enhance insulin resistance and maintain the target level of blood glucose control in addition to weight loss [62]. In the current study, the exercise program was found to significantly decrease, blood glucose level, systolic and diastolic blood pressure and body fat percentage. These findings are consistent with those of the previous studies revealing that exercise in patients with Type 2 diabetes was effective in improving blood glucose control and insulin resistance by promoting the intake and use of blood glucose in the skeletal muscle [63–65].

Therefore, aerobic and resistance workouts are effective in enhancing insulin resistance and lowering blood glucose level in patients with T2DM. In line with our results, Latif et al., [66] found that combination exercises (aerobic plus strength exercise) give a better result in lowering blood glucose level. In the future, combined (aerobic plus strength) exercises can be used as a procedure for regulating and preventing glucose levels in type 2 diabetes mellitus. To support the current result, different research findings also show that selecting one modality or the other may be less important than engaging in any form of physical activity [67]. There are some evidences that a combination of aerobic plus resistance training improves blood glucose control more than either modality alone [68–70].

Patients with T2DM have an excessive threat of atherosclerotic cardiovascular disease, it is important to prevent cardiovascular complications through the management of hypertension, dyslipidemia, and C-reactive proteins (an inflammatory marker) [71]. Exercise has been shown to significantly reduce the risk factors for cardiovascular diseases in patients with T2DM [72]. Combining aerobic and resistance exercise training may reduce blood pressure more effectively than either aerobic exercise or strength training alone [73]. According to Pires et al., [74], the main findings of the study revealed that an acute session of aerobic, resistance, and combined exercises training significantly decreased blood pressure (systolic and diastolic blood pressure). Notably, longer reductions in systolic and diastolic BPs were observed after combined exercise intervention. The three modes of exercise intervention (combined, aerobic and strength exercise intervention) consistently differed in their effects on body composition (body fat percentage and BMI), blood pressure (SBP and DBP) and fasting blood glucose among patients with T2DM.

Multiple exercise training modalities have been recommended, however, it is difficult to determine the superiority of different physical activities for T2DM [33]. Research findings comparing the effect of resistance or aerobic training alone on T2DM and its risk factors but limited studies have compared aerobic, resistance and a combination of this training [34]. The significance of our trial is determined the superiority of different physical activities for T2DM (aerobic, resistance and combined exercise). Here, we found that combined aerobic plus resistance exercise was the superiority of aerobic, resistance alone in enhancing blood glucose level, body composition and blood pressure control for patients with T2DM.

Strength and limitation of this study: the randomization, which ensured that participants were comparable in major study variables at baseline, orientation sessions to minimize potential dropout, which resulted in high attendance are strengths of this study. In addition, in contrast to previous studies [75], all exercise sessions were conducted for the same amount of time, and objective verification of the amount and intensity of exercise performed was carried out. The possibility of over- or under-reporting was eliminated by the objective measurement of all parameters in this study. In addition, physically inactive patients with T2DM who agreed to take part in this study found the exercise instructions to be well tolerated and the participants were both men and women. There are some limitations to this study. One limitation stems from the fact that the findings cannot be applied to other general populations. Our findings are based on a small sample size, and diet intervention was not considered. Future research should consider those limitations.

## Conclusion

The current study findings support the undeniable benefits of regular exercise in patients with T2DM. Generally, aerobic exercise and resistance training alone have positive effects in the prevention or management of blood glucose control and cardiovascular risk factors (blood pressure). Moreover, these effects may be additive in the combination of aerobic plus strength exercise training. Body composition (BMI and body fat percentage), blood pressure (SBP and DBP) and fasting blood glucose significantly decreased in combined (aerobic plus strength) or alone, suggesting that combined (aerobic plus strength) exercise intervention was more effective in changing these measures. Therefore combined aerobic plus resistance exercise was found to be the most effective in enhancing blood glucose level, body composition and blood pressure control for patients with T2DM.

### Electronic supplementary material

Below is the link to the electronic supplementary material.

Supplementary Material 1

## Data Availability

The datasets generated and/or analyzed during the current study are not publicly available due to data security before publication but are available from the corresponding author on reasonable request.

## References

[1] Care A. *2. Classification and diagnosis of diabetes* 2015. 38(Supplement 1): p. S8-S16. 10.2337/dc15-S005 25537714

[2] Federation I (2013). IDF Diabetes Atlas.

[3] Shaw JE et al. *Global estimates of the prevalence of diabetes for 2010 and 2030* 2010. 87(1): p. 4–14. 10.1016/j.diabres.2009.10.007 19896746

[4] A., T., *Diabetes and the public’s health* The Lancet 2009. 374: p. 1140–1. 10.1016/s0140-6736(09)61730-x 19810203

[5] Chan RS (2010). Prevention of overweight and obesity: how effective is the current public health approach. Int J Environ Res Public Health.

[6] Prevention C. f.D.C.a., *National Diabetes Statistics Report. Atlanta (GA): U.S. Department of Health and Human Services* 2014.

[7] Bantie GM et al. *Prevalence of undiagnosed Diabetes Mellitus and associated factors among adult residents of Bahir Dar City, northwest Ethiopia: a community-based cross-sectional study*. 2019. 9(10): p. e030158. 10.1136/bmjopen-2019-030158 PMC6830649 31676649

[8] Bishu KG et al. *Diabetes in Ethiopia: a systematic review of prevalence, risk factors, complications, and cost* 2019. 15: p. 100132.

[9] Sullivan PW et al. *Obesity, inactivity, and the prevalence of Diabetes and diabetes-related cardiovascular comorbidities in the US, 2000–2002*. 2005. 28(7): p. 1599–603. 10.2337/diacare.28.7.1599 15983307

[10] Sudeck G, O.J.A.P H, Höner, Well-Being. *Volitional interventions within cardiac exercise therapy (VIN‐CET): long‐term effects on physical activity and health‐related quality of life*. 2011. 3(2): p. 151–71.

[11] Kohl HW (2012). The Pandemic of Physical Inactivity: Global Action for Public Health.

[12] King N, Hills A, J.J.E.J.o.S S, Blundell. *High Body Mass Index is not a barrier to physical activity: Analysis of international rugby players’ anthropometric data* 2005. 5(2): p. 73–75.

[13] Lindström M, Isacsson S-O, J.J.T.E.J.o.P H, Merlo. *Increasing prevalence of overweight, obesity and Physical Inactivity: two population-based studies 1986 and 1994*. 2003. 13(4): p. 306–12. 10.1093/eurpub/13.4.306 14703316

[14] Klein S (2004). Weight management through lifestyle modification for the prevention and management of type 2 Diabetes: rationale and strategies. A statement of the American Diabetes Association, the North American Association for the Study of Obesity, and the American Society for Clinical. Nutrition.

[15] Snowling NJ, W.G.J.D.c., Hopkins. *Effects of different modes of exercise training on glucose control and risk factors for Complications in type 2 diabetic patients: a meta-analysis*. 2006. 29(11): p. 2518–27. 10.2337/dc06-1317 17065697

[16] Umpierre D et al. *Physical activity advice only or structured exercise training and association with HbA1c levels in type 2 diabetes: a systematic review and meta-analysis* 2011. 305(17): p. 1790–1799. 10.1001/jama.2011.576 21540423

[17] Stone NJ, Saxon D (2005). Approach to treatment of the patient with metabolic syndrome: lifestyle therapy. Am J Cardiol.

[18] Bird SR, Hawley JA (2017). Update on the effects of physical activity on insulin sensitivity in humans. BMJ open Sport & Exercise Medicine.

[19] Najafipour F et al. *Effect of regular exercise training on changes in HbA1c*, *BMI and VO*. 10.1136/bmjdrc-2017-000414 PMC5687538 29177050

[20] Mersy DJ (1991). Health benefits of aerobic exercise. Postgrad Med.

[21] Schwingshackl L (2014). Impact of different training modalities on glycaemic control and blood lipids in patients with type 2 Diabetes: a systematic review and network meta-analysis. Diabetologia.

[22] Cai H (2017). Effect of exercise on the quality of life in type 2 Diabetes Mellitus: a systematic review. Qual Life Res.

[23] Church TS (2004). Exercise capacity and body composition as predictors of mortality among men with Diabetes. Diabetes Care.

[24] Association AD (2017). 2. Classification and diagnosis of Diabetes. Diabetes Care.

[25] Dunstan DW (2008). Aerobic exercise and resistance training for the management of type 2 Diabetes Mellitus. Nat Clin Pract Endocrinol Metab.

[26] Sullivan PW (2005). Obesity, inactivity, and the prevalence of Diabetes and diabetes-related cardiovascular comorbidities in the US, 2000–2002. Diabetes Care.

[27] Dutton RA, Khadavi MJ, Fredericson M (2014). Update on rehabilitation of patellofemoral pain. Curr Sports Med Rep.

[28] Rydén L et al. *ESC Guidelines on diabetes, pre-diabetes, and cardiovascular diseases developed in collaboration with the EASD: the Task Force on diabetes, pre-diabetes, and cardiovascular diseases of the European Society of Cardiology (ESC) and developed in collaboration with the European Association for the Study of Diabetes (EASD)* Eur Heart J, 2013. 34(39): p. 3035-87. 10.1093/eurheartj/eht108 23996285

[29] Hansen D (2013). Exercise assessment and prescription in patients with type 2 Diabetes in the private and home care setting: clinical recommendations from AXXON (Belgian Physical Therapy Association). Phys Ther.

[30] Colberg SR (2010). Exercise and type 2 Diabetes: the American College of Sports Medicine and the American Diabetes Association: joint position statement. Diabetes Care.

[31] Hordern MD (2012). Exercise prescription for patients with type 2 Diabetes and pre-diabetes: a position statement from Exercise and Sport Science Australia. J Sci Med Sport.

[32] Oliveira C (2012). Combined exercise for people with type 2 Diabetes Mellitus: a systematic review. Diabetes Res Clin Pract.

[33] Pan B (2018). Exercise training modalities in patients with type 2 Diabetes Mellitus: a systematic review and network meta-analysis. Int J Behav Nutr Phys Act.

[34] Ho SS (2012). The effect of 12 weeks of aerobic, resistance or combination exercise training on cardiovascular risk factors in the overweight and obese in a randomized trial. BMC Public Health.

[35] Liguori G, Medicine ACoS. ACSM’s guidelines for exercise testing and prescription. Lippincott Williams & Wilkins; 2020.

[36] Brown LN. The effects of a 5-week nutrition education intervention on collegiate athletes’ knowledge and dietary intake. Oklahoma State University; 2009.

[37] Medicine ACoS. *ACSM’s (2018). Guidelines for Exercise Testing and prescription*. Philadelphia: Lippincott Williams and Wilkins.

[38] Medicine ACoS. ACSM’s health-related physical fitness assessment manual. Lippincott Williams & Wilkins; 2013.

[39] Jackson AS, Pollock ML (1985). Practical assessment of body composition. The Physician and Sportsmedicine.

[40] Regassa IF et al. *Development and validation of food frequency questionnaire for food and nutrient intakes of adults in Butajira, southern Ethiopia*. J Nutritional Sci, 2021. 10. 10.1017/jns.2021.94 PMC8635871 34888036

[41] Aneja O. *Warming-up, cooling down–meaning and significance*. Eur J Mol Clin Med. 7: p. 5263–5.

[42] Pescatello L (2004). Exercise and Hypertension. Medicine&science in sports&exercise. J Amer Coll Sports Med.

[43] Garber CE et al. *Quantity and quality of exercise for developing and maintaining cardiorespiratory, musculoskeletal, and neuromotor fitness in apparently healthy adults: guidance for prescribing exercise* 2011. 10.1249/MSS.0b013e318213fefb 21694556

[44] Gamboa H, Fred A. *A behavioral biometric system based on human-computer interaction*. In *Biometric Technology for Human Identification*. SPIE; 2004.

[45] Gellish RL (2007). Longitudinal modeling of the relationship between age and maximal heart rate. Med Sci Sports Exerc.

[46] Carvalho CJd (2019). Aerobic and resistance exercise in patients with resistant Hypertension. Revista Brasileira De Medicina do Esporte.

[47] Borg GA. Psychophysical bases of perceived exertion. Medicine & science in sports & exercise; 1982. 7154893

[48] Smith JR (2015). Does menstrual cycle phase affect lung diffusion capacity during exercise?. Respir Physiol Neurobiol.

[49] Constantini NW, Dubnov G, Lebrun CM (2005). The menstrual cycle and sport performance. Clin Sports Med.

[50] Julian R (2017). The effects of menstrual cycle phase on physical performance in female soccer players. PLoS ONE.

[51] Leslie JR, Stephens MA, Fotopoulos S. Asymptotic distribution of the Shapiro-Wilk $W$ for testing for normality. Volume 14. The Annals of Statistics; 1986. pp. 1497–506. 4.

[52] Cohen J, et al. Applied multiple regression/correlation analysis for the behavioral sciences. Routledge; 2013.

[53] Mendes R et al. *Exercise prescription for patients with type 2 diabetes—a synthesis of international recommendations: narrative review*. 2016. 50(22): p. 1379–81. 10.1136/bjsports-2015-094895 26719499

[54] Members ATF et al. *ESC Guidelines on diabetes, pre-diabetes, and cardiovascular diseases developed in collaboration with the EASD: the Task Force on diabetes, pre-diabetes, and cardiovascular diseases of the European Society of Cardiology (ESC) and developed in collaboration with the European Association for the Study of Diabetes (EASD)* 2013. 34(39): p. 3035–3087. 10.1093/eurheartj/eht108 23996285

[55] Colberg SR et al. *Exercise and type 2 Diabetes: the American College of Sports Medicine and the American Diabetes Association: joint position statement*. 2010. 33(12): p. e147–67. 10.2337/dc10-9990 PMC2992225 21115758

[56] Hansen D et al. *Exercise assessment and prescription in patients with type 2 Diabetes in the private and home care setting: clinical recommendations from AXXON (Belgian Physical Therapy Association)*. 2013. 93(5): p. 597–610. 10.2522/ptj.20120400 23392184

[57] Mendes R (2016). Exercise prescription for patients with type 2 diabetes—a synthesis of international recommendations: narrative review. Br J Sports Med.

[58] Kang S-J, Ko K-J, Baek U-H (2016). Effects of 12 weeks combined aerobic and resistance exercise on heart rate variability in type 2 Diabetes Mellitus patients. J Phys Therapy Sci.

[59] Park S-K (2003). The effect of combined aerobic and resistance exercise training on abdominal fat in obese middle-aged women. J Physiol Anthropol Appl Hum Sci.

[60] Keles A (2007). The effects of the priority in the aerobic training and strength training combination for burning fat in a workout.

[61] Poehlman ET (2000). Effects of resistance training and endurance training on insulin sensitivity in nonobese, young women: a controlled randomized trial. J Clin Endocrinol Metabolism.

[62] Walker K et al. *Diet and exercise in the prevention of Diabetes*. 2010. 23(4): p. 344–52. 10.1111/j.1365-277X.2010.01061.x 20337844

[63] Cuff DJ et al. *Effective exercise modality to reduce insulin resistance in women with type 2 Diabetes*. 2003. 26(11): p. 2977–82. 10.2337/diacare.26.11.2977 14578226

[64] Liu Y et al. *Effects of combined aerobic and resistance training on the glycolipid metabolism and inflammation levels in type 2 diabetes mellitus* 2015. 27(7): p. 2365–2371. 10.1589/jpts.27.2365 PMC4540883 26311110

[65] Cauza E et al. *The relative benefits of endurance and strength training on the metabolic factors and muscle function of people with type 2 Diabetes Mellitus*. 2005. 86(8): p. 1527–33. 10.1016/j.apmr.2005.01.007 16084803

[66] Latif S, Utomo DN, Rejeki PS. *Combination of Aerobic and Resistance Exercise in Lowering Blood Glucose Levels Compared to Aerobic or Resistance Exercises in a Male Wistar Rat Model with Diabetes Mellitus* 2017.

[67] Yang Z et al. *Resistance exercise versus aerobic exercise for type 2 diabetes: a systematic review and meta-analysis* 2014. 44(4): p. 487–499. 10.1007/s40279-013-0128-8 24297743

[68] Church TS et al. *Effects of aerobic and resistance training on hemoglobin A1c levels in patients with type 2 Diabetes: a randomized controlled trial*. 2010. 304(20): p. 2253–62. 10.1001/jama.2010.1710 PMC3174102 21098771

[69] D’hooge R et al. *Influence of combined aerobic and resistance training on metabolic control, cardiovascular fitness and quality of life in adolescents with type 1 Diabetes: a randomized controlled trial*. 2011. 25(4): p. 349–59. 10.1177/0269215510386254 21112904

[70] Sigal RJ et al. *Effects of aerobic training, resistance training, or both on glycemic control in type 2 Diabetes: a randomized trial*. 2007. 147(6): p. 357–69. 10.7326/0003-4819-147-6-200709180-00005 17876019

[71] Adler AI et al. *Association of systolic blood pressure with macrovascular and microvascular Complications of type 2 Diabetes (UKPDS 36): prospective observational study*. 2000. 321(7258): p. 412–9. 10.1136/bmj.321.7258.412 PMC27455 10938049

[72] Marwick TH et al. *Exercise training for type 2 diabetes mellitus: impact on cardiovascular risk: a scientific statement from the American Heart Association* 2009. 119(25): p. 3244–3262. 10.1161/CIRCULATIONAHA.109.192521 19506108

[73] Caminiti G et al. *Effects of 12 weeks of aerobic versus combined aerobic plus resistance exercise training on short-term blood pressure variability in patients with Hypertension*. 2021. 130(4): p. 1085–92. 10.1152/japplphysiol.00910.2020 33630677

[74] Pires NF et al. *Combined aerobic and resistance exercises evokes longer reductions on ambulatory blood pressure in resistant hypertension: a randomized crossover trial* 2020. 2020. 10.1155/2020/8157858 PMC7416229 32821284

[75] Sigal RJ (2007). Effects of aerobic training, resistance training, or both on glycemic control in type 2 Diabetes: a randomized trial. Ann Intern Med.

