# Supplementary material for: The impact of exercise modalities on blood glucose, blood pressure and body composition in patients with type 2 diabetes mellitus
Source: BMC Sports Sci Med Rehabil. 2023 Nov 14;15:153. doi: 10.1186/s13102-023-00762-9 (PMC10644520; doi:10.1186/s13102-023-00762-9)
Supplement: Supplementary file 1 — Supplementary Material 1 [file 13102_2023_762_MOESM1_ESM.docx]

Table S1: Tests of between-subjects effects

| BMI | Source | Type III  squares | Df | Mean square | F | Sig. | Partial Eta Squared |
| --- | --- | --- | --- | --- | --- | --- | --- |
|  | Corrected Model | 201.647a | 6 | 33.608 | 6.790 | .000 | .552 |
|  | Intercept | 30.663 | 1 | 30.663 | 6.195 | .018 | .158 |
|  | Diet | .231 | 1 | .231 | .047 | .830 | .001 |
|  | Gender | 2.981 | 1 | 2.981 | .602 | .443 | .018 |
|  | Age | 11.158 | 1 | 11.158 | 2.254 | .143 | .064 |
|  | Aerobic exercise IG | 175.156 | 3 | 58.385 | 11.796 | .000 | .517 |
|  | Error | 163.343 | 33 | 4.95 |  |  |  |
|  | Total | 163.343 | 21277.78 | 40 |  |  |  |
|  | Corrected Total | 364.990 | 39 |  |  |  |  |
| FBG | Corrected Model | 2373.149b | 6 | 395.525 | 3.759 | .006 | .406 |
|  | Intercept | 822.287 | 1 | 822.287 | 7.814 | .009 | .191 |
|  | Diet | 14.953 | 1 | 14.953 | .142 | .709 | .004 |
|  | Gender | .490 | 1 | .490 | .005 | .946 | .000 |
|  | Age | .003 | 1 | .003 | .000 | .996 | .000 |
|  | Aerobic exercise IG | 2000.424 | 3 | 666.808 | 6.337 | .002 | .366 |
|  | Error | 3472.626 | 33 | 105.231 |  |  |  |
|  | Total | 785093.000 | 40 |  |  |  |  |
|  | Corrected Total | 5845.775 | 39 |  |  |  |  |
| SBP | Corrected Model | 4332.941c | 6 | 722.157 | 7.322 | .000 | .571 |
|  | Intercept | 1171.084 | 1 | 1171.084 | 11.874 | .002 | .265 |
|  | Diet | .065 | 1 | .065 | .001 | .980 | .000 |
|  | Gender | .255 | 1 | .255 | .003 | .960 | .000 |
|  | Age | 87.276 | 1 | 87.276 | .885 | .354 | .026 |
|  | Aerobic exercise IG | 3959.826 | 3 | 1319.942 | 13.383 | .000 | .549 |
|  | Error | 3254.659 | 33 | 98.626 |  |  |  |
|  | Total | 735508.000 | 40 |  |  |  |  |
|  | Corrected Total | 7587.600 | 39 |  |  |  |  |
| DBP | Corrected Model | 3033.688d | 6 | 505.615 | 5.731 | .000 | .510 |
|  | Intercept | 91.784 | 1 | 91.784 | 1.040 | .315 | .031 |
|  | Diet | 103.585 | 1 | 103.585 | 1.174 | .286 | .034 |
|  | Gender | 111.080 | 1 | 111.080 | 1.259 | .270 | .037 |
|  | Age | 94.080 | 1 | 94.080 | 1.066 | .309 | .031 |
|  | Aerobic exercise IG | 2072.457 | 3 | 690.819 | 7.830 | .000 | .416 |
|  | Error | 2911.599 | 33 | 88.230 |  |  |  |
|  | Total | 327555.710 | 40 |  |  |  |  |
|  | Corrected Total | 5945.288 | 39 |  |  |  |  |
| BFP | Corrected Model | 1322.411e | 6 | 220.402 | 13.683 | .000 | .713 |
|  | Intercept | 47.800 | 1 | 47.800 | 2.967 | .094 | .083 |
|  | Diet | .282 | 1 | .282 | .018 | .896 | .001 |
|  | Gender | 2.306 | 1 | 2.306 | .143 | .708 | .004 |
|  | Age | 5.034 | 1 | 5.034 | .312 | .580 | .009 |
|  | Aerobic exercise IG | 1173.966 | 3 | 391.322 | 24.293 | .000 | .688 |
|  | Error | 531.571 | 33 | 16.108 |  |  |  |
|  | Total | 38133.523 | 40 |  |  |  |  |
|  | Corrected Total | 1853.982 | 39 |  |  |  |  |
